# Hospitals in India

**Published:** 1893-11-04

**Authors:** 


### HOSPITALS IN INDIA.

### I.—THE BENGAL PRESIDENCY.

#### 4. Dispensaries in the Central Provinces.

At the close of 1891 there were 111 dispensaries existing in the Central Provinces, or four more than in the previous year, and the number of available beds was 607 for males and 287 for females. The attendance of both indoor and outdoor patients showed an increase, having been 8,222 and 904,654 in 1891 as compared with 7,885 and 867,219 respectively in 1890. Variations in the number of indoor patients are due chiefly to the larger or smaller number of destitute persons brought in by the police in a moribund condition. Many persons thus brought in are beggars, poverty-stricken wanderers from the North-Western Provinces or from Central India, coolies in search of employment, and the like. The result is that the death-rate among the indoor patients is frequently high. The provincial ratio of deaths to total treated was 11·88 in 1891, as compared with 10·07 in 1890, the highest percentage being 21 at Bilaspur.

It is recognised that the large number of deaths tends to discredit a hospital and depress patients, and efforts are made to provide separate accommodation for these moribund waifs and strays. The Victoria Hospital, Jubbulpore, has a "moribund" ward, and a "poor house" for a similar purpose exists at Damoh.

Of the total of 8,222 indoor patients, representing a daily average number of 365·02, 5,655 were cured, 693 were relieved, 562 were discharged "otherwise," and 977 died. With regard to major surgical operations, those performed by the superintendents numbered 535, and the results were 418 cures, 34 discharges relieved, 32 discharges "otherwise," and 31 deaths. In addition to these 851 major operations were performed by the medical subordinates, and 741 patients were cured, 44 relieved, 18 discharged "otherwise," and 22 died. The year shows an increase of 166 surgical operations, or just over 14 per cent. more than in 1890.

The total revenue of the dispensaries was Rs.1,50,075, as against Rs.1,44,012 in the previous year. Government provided Rs.46,649, local funds Rs.7,838, municipal funds Rs.57,430, European subscriptions Rs.5,202, native subscriptions Rs.23,914, and the rest came from interest on investments, sale of securities, and surplus from the previous year.

The gross expenditure amounted to Rs.1,43,449, of which Rs.69,217 went on establishments, Rs.2,788 on bazaar medicines, Rs.24,447 on European medicines, Rs.8,968 on diets, Rs.9,091 on miscellaneous charges, and Rs.23,604 on buildings and repairs. There was a cash balance left of Rs.6,626 at the end of the year. The average cost of each diet was 1a. 6p., and the percentage of the total cost paid by Government was 33·77. Regarded as a whole this report of the dispensaries in the Central Provinces for 1891 must be considered satisfactory.

